# Role of visceral fat areas defined by thoracic CT in acute pulmonary embolism

**DOI:** 10.1259/bjr.20211267

**Published:** 2022-03-18

**Authors:** Hans-Jonas Meyer, Franz Benkert, Nikolaos Bailis, Marianne Lerche, Alexey Surov

**Affiliations:** Department of Diagnostic and Interventional Radiology, University of Leipzig, Leipzig, Germany; Department of Diagnostic and Interventional Radiology, University of Leipzig, Leipzig, Germany; Department of Diagnostic and Interventional Radiology, University of Leipzig, Leipzig, Germany; Department of Respiratory Medicine, University Hospital Leipzig, University of Leipzig, Leipzig, Germany; Department of Radiology and Nuclear Medicine, Otto von Guericke University, Magdeburg, Germany

## Abstract

**Objective:**

Visceral adipose tissue (VAT) has been established as an important parameter of body composition. It can be assessed by imaging modalities like computed tomography (CT). The purpose of the present study was to analyse the prognostic role of VAT derived from thoracic CT in patients with acute pulmonary embolism (PE).

**Methods:**

The clinical database of our center was retrospectively screened for patients with acute PE between 2014 and 2017. Overall, 184 patients were included into the analysis. VAT was assessed on axial slices of the thoracic CT at the level of the first lumbar vertebra. Clinical scores, serological parameters, need for intubation, ICU admission and 30 days mortality were assessed.

**Results:**

Using the previously reported threshold of 100 cm² for visceral obesity definition 136 (73.9%), patients were considered as visceral obese. There was a moderate correlation between VAT and BMI (*r* = 0.56, *p* < 0.0001). There was also a moderate correlation between VAT and body height (*r* = 0.41, *p* =< 0.0001). Of all investigated clinical scores relating to acute PE, only the GENEVA score correlated weakly with VAT (*r* = 0.15, *p* = 0.04). There were significant correlations between VAT and creatinine (*r* = 0.38, *p* < 0.0001) and Glomerular filtration rate (*r* = −0.21, *p* = 0.005). No associations were identified for VAT and mortality or visceral obesity and mortality.

**Conclusion:**

VAT was not associated with mortality in patients with acute pulmonary embolism.

**Advances in knowledge:**

Visceral obesity is frequent in patients with acute pulmonary embolism but it is not associated with mortality.

## Introduction

Acute pulmonary embolism (PE) is a possible life-threatening cardiovascular disease with 30-day mortality rates ranging from 0.5% to over 20%.^1,2^ Yet, there are also low-risk clinical courses without severe complications.^2^ Therefore, it is important to perform an immediate risk stratification of patients with acute PE at the time of presentation in clinical routine.

The CT pulmonary angiography (CTPA) is established as the diagnostic clinical gold standard in diagnosis of PE with a reported sensitivity and specificity up to 100%.^3^ It is in most cases the first imaging performed in these patients, at best directly after the admission. Risk stratification based on CTPA could be clinically important.^3–5^ Established CT signs with prognostic relevance in acute PE are right heart strain, dilatation of the right atrium and contrast media influx into the inferior vena cava.^4–7^

Body composition is an emergent research field to assess different fat areas (visceral and subcutaneous (VAT and SAT)) and skeletal muscle areas.^8–11^ The prognostic value of these parameters is of great importance in several fields of medicine. In most studies, a single slice of the abdomen is used, preferably on L3 level.^9,10^

However, the scan range of the CTPA does not the cover the abdomen to this level, which hinders its possibility to estimate reliable VAT parameters in this disorder. Yet, promising studies proposed alternative VAT parameters derived from thoracic CTs.^11–14^ For example, there were promising results for COVID-19 patients that utilised the last slice of thoracic CT to estimate the amount of visceral fat.^11–14^

In these studies, thoracic CT was used to obtain one axial slice on the mid of the first lumbar vertebra. Notably, there were significant associations between VAT and mortality in COVID-19 patients highlighting the prognostic relevance of this new parameter.^11–14^

Although there are promising reports regarding the use of body composition for risk stratification in other conditions, the possible benefit of VAT quantification in patients with PE is still unknown.

Therefore, the purpose of the present study was to analyse the prognostic role of VAT derived from thoracic CT in patients with acute PE.

## Methods and materials

### Patient acquisition

This retrospective study was approved by the institutional review board (Nr: 118/19-ck, Ethics Committee, University of Leipzig, Leipzig, Germany).

All patients with acute PE were retrospectively assessed within the time period 2014 to 2017.

The patient sample is based on a previous study, which investigated the associations between clinical parameters and pulmonary CT obstruction scores in patients with acute PE.^15^

### Clinical parameters

For clinical parameters, the following values were retrieved at the time point of hospital admission: blood pressure (mmHg), heart rate (n/minute), need of intubation, need of vasopressor, need for intensive care admission.

The following clinical scores were calculated: Wells score,^16^ revised Geneva score,^17^ and sPESI score.^18^ Furthermore, D-dimer level (μg/mL), lactate (venous blood, mmol/L), pH (venous blood), troponin C (pg/mL), and N-terminal natriuretic peptide (BNP, pg/mL) were collected.

Mortality was assessed in days after diagnosis of PE. All-cause mortality was assessed in the present study over a time period of 30 days.

### Imaging technique

CTPA was performed on a 128-slice CT scanner (Ingenuity 128, Philips, Hamburg, Germany). Intravenous administration of an iodine-based contrast medium (60 ml Imeron 400 MCT, Bracco Imaging Germany GmbH, Konstanz, Germany) was given at a rate of 4.0 ml s^−1^ via a peripheral venous line. Automatic bolus tracking was performed in the pulmonary trunk with a trigger of 100 Hounsfield units (HU). Typical imaging parameters were: 100 kVp; 125 mAs; slice thickness, 1 mm; pitch, 0.9. In every case, CTPA was performed in deep inspiration level.

### Imaging findings

PE was diagnosed in a retrospective evaluation by an experienced radiologist. PE was only diagnosed, when the contrast-filling defect of the pulmonary artery was seen in at least two slices.

The right heart strain was evaluated with dilation of the right ventricle, measured as the short axis ratio right ventricle/left ventricle (RV/LV).^19^

Contrast media reflux into the vena cava inferior and hepatic veins was graded as follows: 0 = non-existing, 1 = reflux into the vena cava inferior, 2 = reflux into the hepatic veins, 3 = reflux into subhepatic vena cava inferior, according to a previous investigation.^7^

The total embolus burden was quantified according to the established Mastora score.^20^ This scoring system includes five mediastinal, six lobar, and 20 segmental arteries, which are each scored for the degree of embolus material obstruction on a scale from 0 to 5 (0 = 0 %, 1 = 1–24%, 2 = 25–49%, 3 = 50–74%, 4 = 75–99%, 5 = 100 %). The sum of mediastinal, lobar and segmental artery scores lead to a global obstruction score with a maximum of 155.

### VAT quantification

Visceral fat areas were semiautomatically measured with the freely available ImageJ software 1.48v (National Institutes of Health Image program). One axial slice on the mid of the first lumbar vertebral (L1) was used, as was used in a recent study investigating the effects between mortality and VAT in COVID-19 patients.^14^ The visceral fat area was semiautomatically measured using the HU threshold levels of −190 and −30 HU.^21^

**Figure 1.**
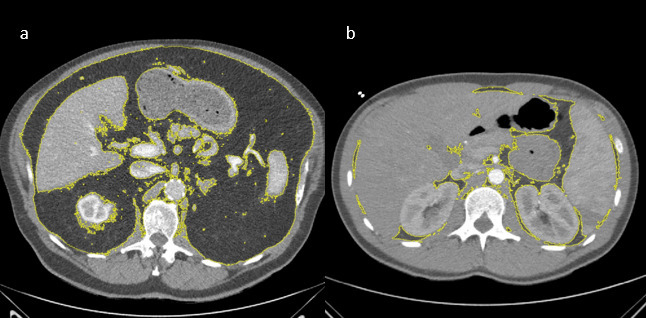
(**a**)Representative case of the patient sample with high amount of visceral fat and acute PE. The axial slice on level of the mid of the first lumbar vertebra with ROI drawn to measure the whole amount of visceral fat of this slice. (**b**)Another representative case of the patient sample with low amount of visceral fat with drawn region of interest.

We used the proposed threshold value of 100 cm² as a cut-off value to determine visceral obesity, as it was performed in a recent study by Goehler et al in thoracic CT images.^12^ Figure 1 displays two representative patients with the VAT calculation for illustrative purposes.

### Statistical analysis

The statistical analysis and graphics creation were performed using GraphPad Prism (GraphPad Software, La Jolla, CA, USA) and SPSS STATISTICS (IBM, Version 25.0; Armonk, NY, USA). Collected data were evaluated by means of descriptive statistics (absolute and relative frequencies). Spearman’s correlation coefficient (r) was used to analyse associations between the investigated scores after testing for normality distribution. Group differences were calculated with Mann-Whitney test and Fisher exact test, when suitable. Kaplan-Meier curves were used for survival analysis. Multivariate Cox regression were used to test for the effect of VAT on mortality. Possible known confounding factors for visceral obesity, such as age, gender and BMI were used for the multivariate analysis. In all instances, *p-v*alues < 0.05 were taken to indicate statistical significance.

## Results

Overall, 286 patients were screened. After exclusion due to insufficient clinical and imaging data, 184 patients (89 female patients, 48.3%) with a mean age of 65.1 ± 16.3 years, range 19–100 years were identified in the data base and included into the present study. Consequently, patients with chronic PE were not included into the study.

### Severity of PE

Overall, 48 patients (26.1%) of the patient sample died with a median of 1.8 days, range 1–60 days. Only 3 patients (6.3% of the non-survivors) died after 30 days and were not included into the analysis. Therefore, 45 patients were included into the 30 days mortality analysis. Furthermore, 144 patients (78.2%) were admitted to the intensive care unit (ICU).

The mean VAT was 176.4 cm²±110.4, range 15.2–545.3 cm². According to the threshold-value of 100 cm², 136 patients (73.9%) were considered as visceral obese and 48 patients (26.1%) as not.

Clinically, systolic blood pressure, venous lactate and pH were significantly different between survivors and non-survivors. SPESI score values were higher in non-survivors compared to survivors (*p* < 0.0001).

Table 1 displays the comparison between visceral obese and lean patients. Male gender was highly associated with visceral obesity. As expected, BMI was significantly higher in visceral obese patients compared to lean patients.

**Table 1.** Comparison between visceral obese and lean patients

Parameter	Visceral Obese (*n* = 136)	Lean (*n* = 48)	*p*-value
Age (y)	65.9 ± 13.6	62.6 ± 22.0	0.92
Gender (female, n, %)	48 (35.3)	41 (85.4)	**0.0015^*a*^**
Systolic blood pressure (mmHg)	124.6 ± 29.2	122.5 ± 28.0	0.64
Heart rate (pulse/min)	106 ± 22	102 ± 23	0.28
Respiratory rate (1 /min)	23 ± 8	24 ± 8	0.99
BMI (kg/m²)	29.5 ± 6.1	24.3 ± 4.0	**<0.0001**
Troponin c (ng/ml)	82.5 ± 110.6	64.7 ± 65.4	0.29
D-Dimer (ng/ml)	11.3 ± 9.3	18.6 ± 27.1	0.59
Venous pH	7.3 ± 0.1	7.3 ± 0.2	0.54
Venous lactate (mmol/L)	3.1 ± 2.8	3.6 ± 3.3	0.99
Wells score	6.1 ± 1.8	6.0 ± 2.0	0.48
GENEVA score	7.8 ± 3.5	7.4 ± 3.6	0.42
sPESI score	2.1 ± 1.1	2.3 ± 1.3	0.63
Intubation (n, %)	46 (33.8)	16 (33.3)	0.99^*a*^
ICU admission (n, %)	106 (72.6)	38 (79.2)	0.99^*a*^
Mortality (n, %)	36 (26.5)	12 (25)	0.99^*a*^
Mastora score	80.9 ± 24.4	72.9 ± 28.5	0.12
VAT (cm²)	218.3 ± 98.1	57.9 ± 20.9	**<0.0001**

BMI, Body mass index; ICU, intensive care unit; VAT, visceral adipose tissue.

Significant p-values are highlighted in bold.

a Fishers-Exact test.

### Association between visceral obesity and mortality

**Figure 2.**
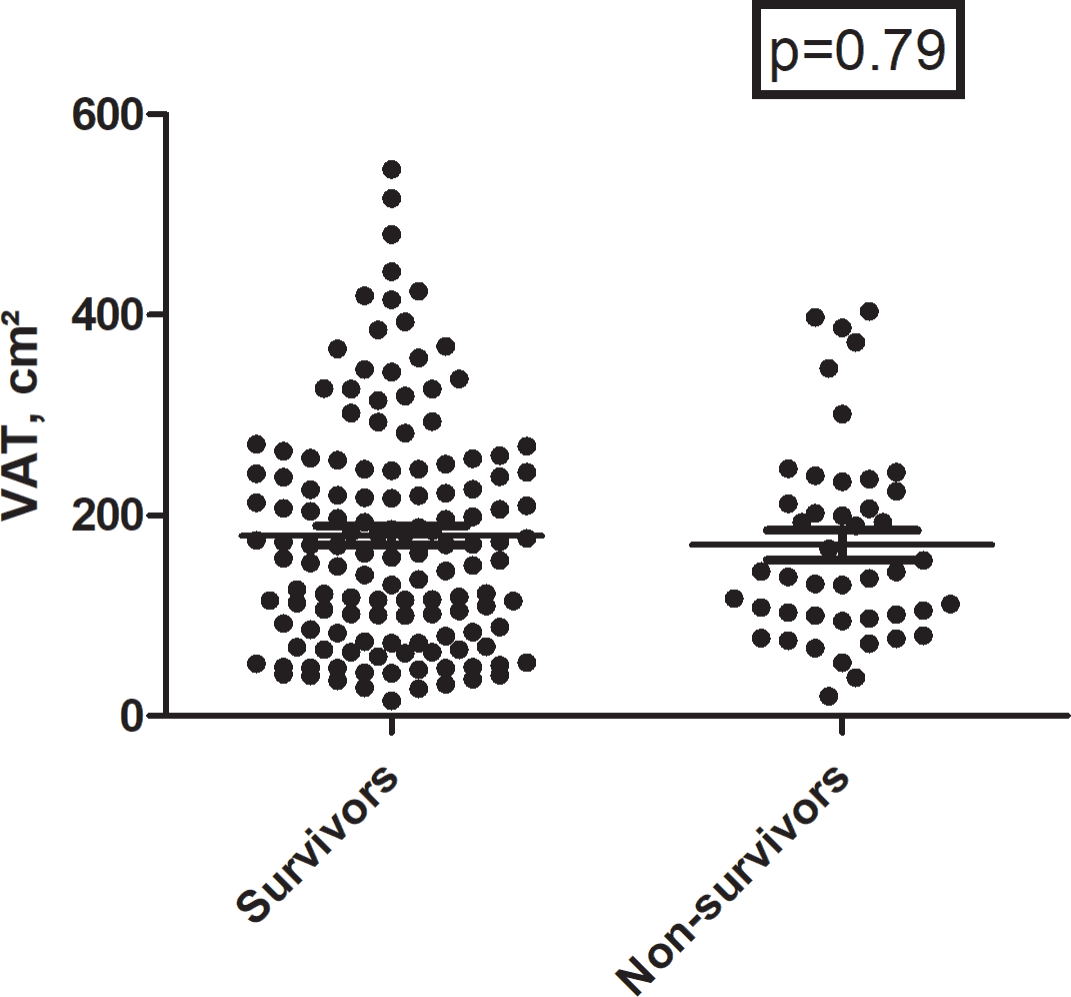
Scatter plot of VAT values between survivors and non-survivors. There was no statistically significant difference between the groups (*p* = 0.79).

**Figure 3.**
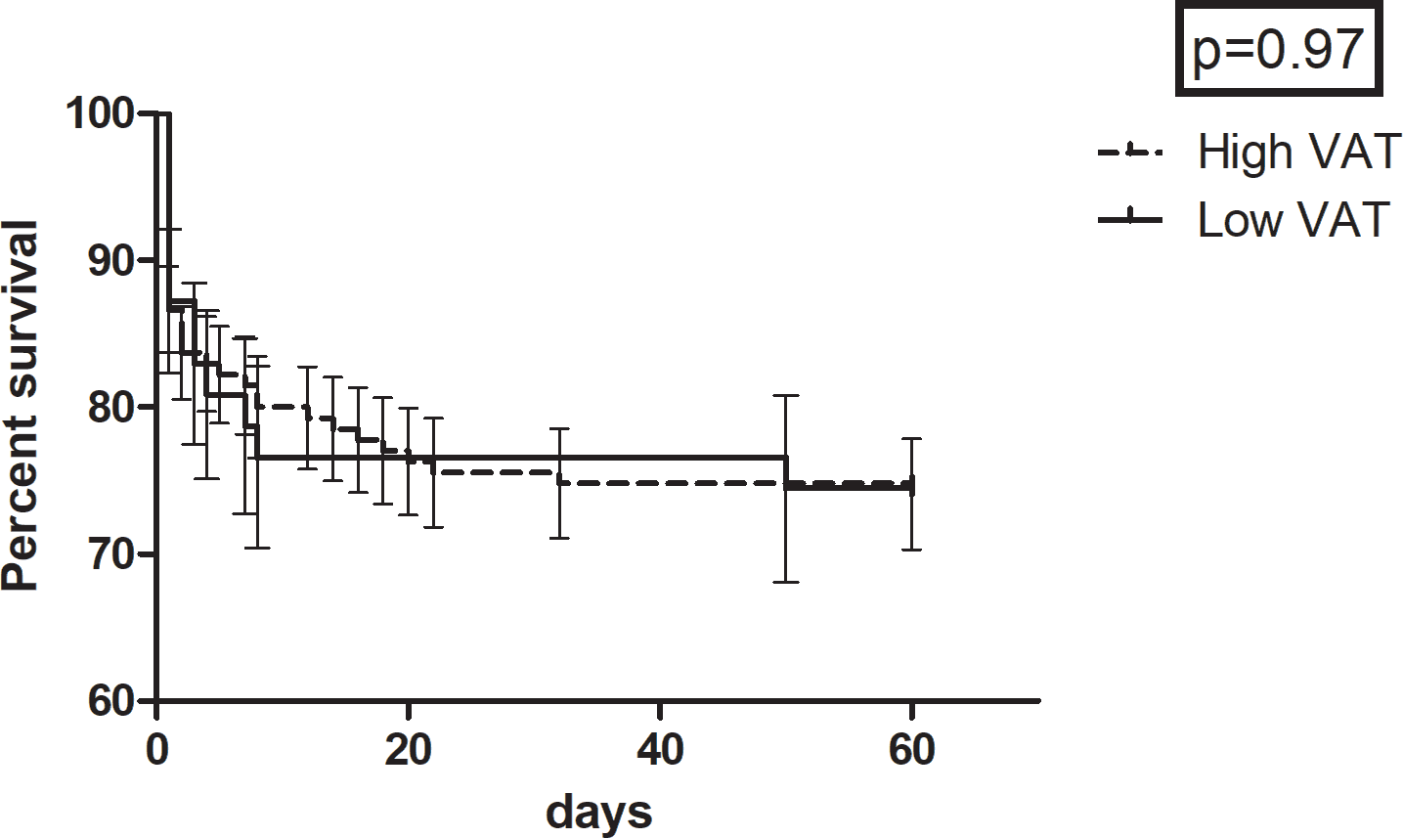
Kaplan-Meier curve of the association between visceral obesity and mortality. There were no significant differences in regard of mortality for patients with visceral obesity defined by the threshold value of 100 cm² and those without (*p* = 0.97).

**Figure 4.**
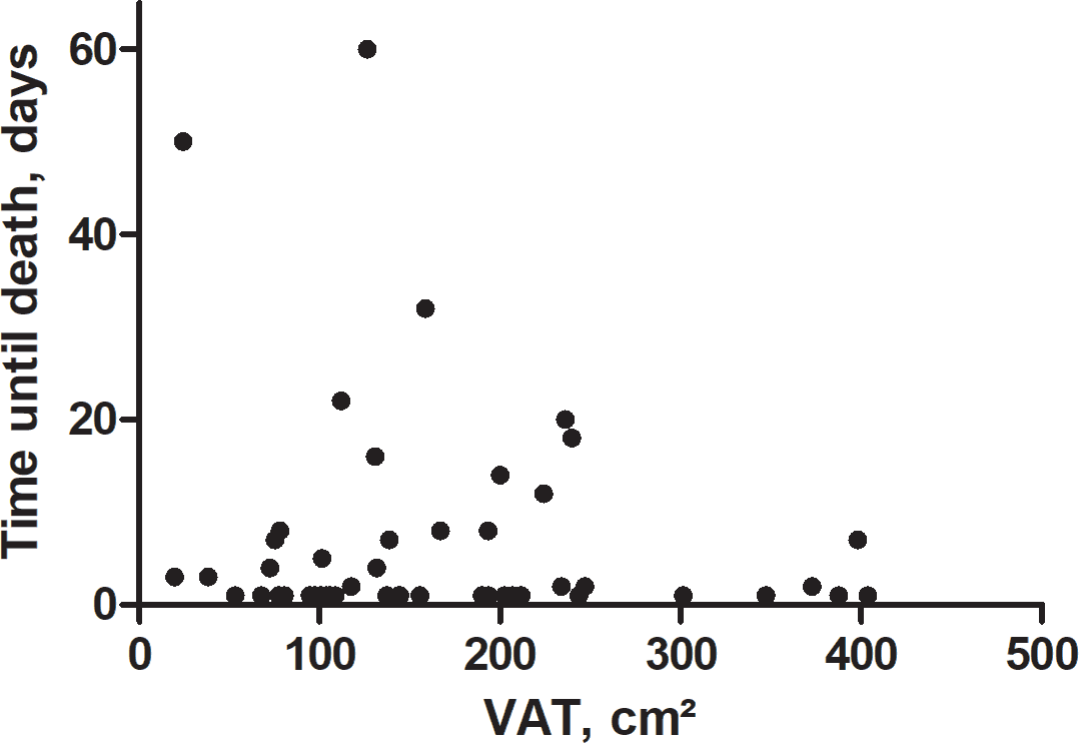
Duration of the survival of the patients. There was no association with VAT (*r* = −0.04, *p* = 0.77).

There were no differences in regard to VAT and frequency of visceral obesity between survivors and non-survivors (Figure 2). Also, there were no significant differences in regard of mortality for patients with and without visceral obesity defined by the threshold value of 100 cm² (*p* = 0.97). The Kaplan-Meier curve is displayed by Figure 3. Figure 4 displays the survival duration of the patients in association with VAT (*r* = −0.04, *p* = 0.77).

In multivariate Cox regression, VAT, age, gender, and BMI had no influence on mortality (Table 2).

**Table 2.** Cox regression analysis for death

Variable	Hazard Ratio (95% CI)	*p*-value
VAT	1.0 (0.99–1.004)	0.62
Age	1.01 (0.98–1.03)	0.36
Gender	0.82 (0.43–1.59)	0.57
BMI	1.02 (0.96–1.08)	0.48

BMI, body mass index; CI, confidence interval; VAT, visceral adipose tissue.

### Correlations between VAT and clinical, serological, and radiological parameters

**Figure 5.**
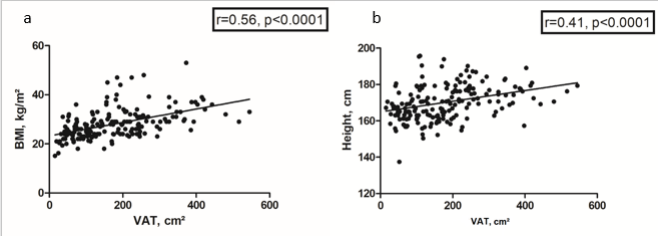
(a) Spearman’s correlation analysis between VAT and BMI. The correlation coefficient is 0.56, *p* < 0.0001. (**b**). Spearman’s correlation analysis between VAT and body height. The correlation coefficient is 0.41, *p* < 0.0001.

There was a moderate correlation between VAT and BMI (*r* = 0.56, *p* < 0.0001), Figure 5a. Also, VAT correlated moderately with body height (*r* = 0.41, *p* =< 0.0001) (Figure 5b).

**Figure 6.**
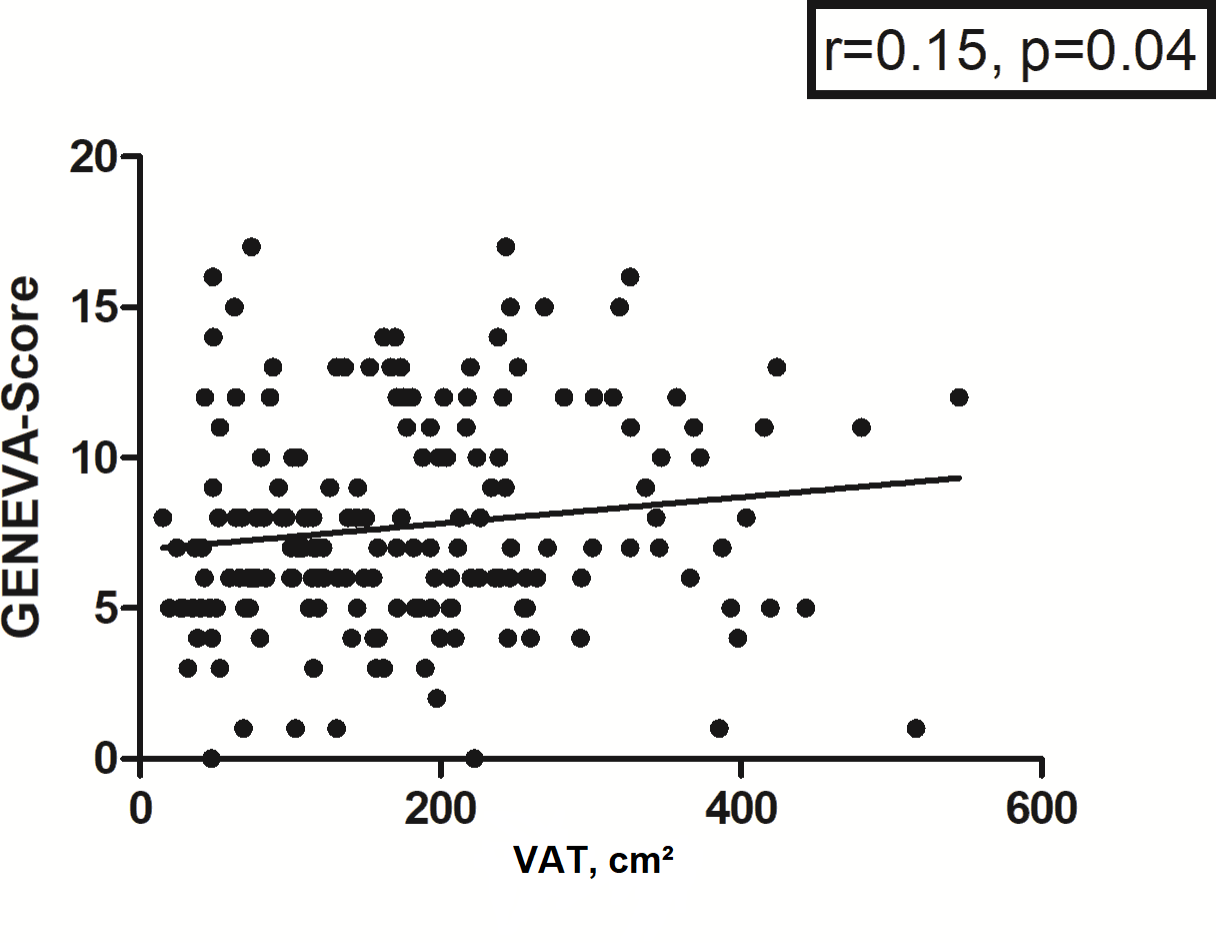
Spearman’s correlation analysis between VAT and GENEVA-Score. The correlation coefficient is 0.14, *p* = 0.04.

Of all investigated clinical scores, only the Geneva score correlated weakly with VAT (*r* = 0.15, *p* = 0.04) (Figure 6).

**Figure 7.**
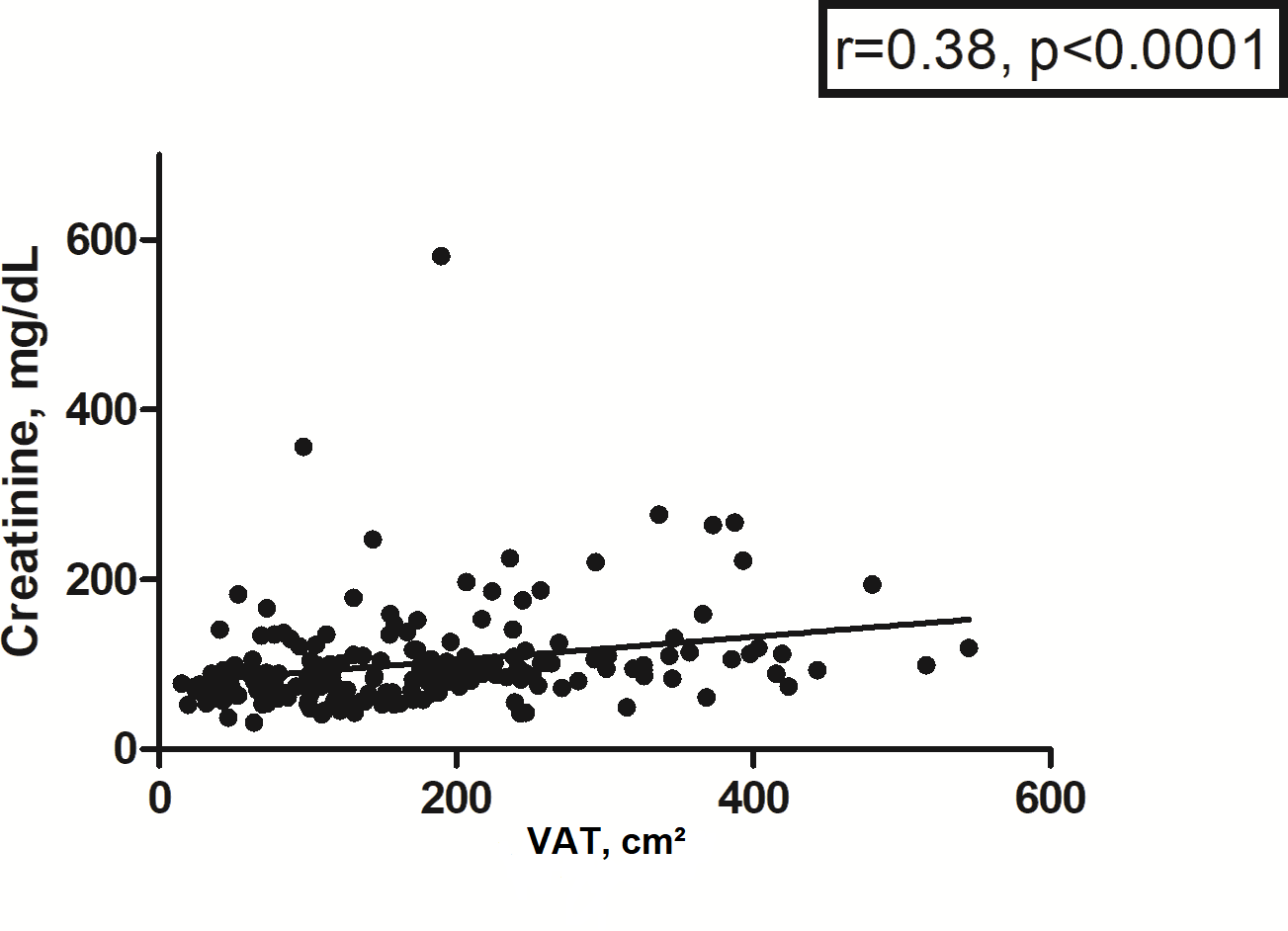
Spearman’s correlation analysis between VAT and creatinine. The correlation coefficient is 0.38, *p* < 0.0001.

VAT correlated also with serum creatinine (*r* = 0.38, *p* < 0.0001) and glomerular filtration rate (*r* = −0.21, *p* = 0.005) (Figure 7). Finally, VAT correlated with haematocrit (*r* = 0.21, *p* = 0.003) and haemoglobin (*r* = 0.22, *p* = 0.002). In the visceral obese group alone, there was a weak association between VAT and venous lactate (*r* = 0.21, *p* = 0.04).

The other investigated parameters did not correlate significantly with VAT.

VAT did not correlate with reflux into the vena cava inferior (*r* = −0.04, *p* = 0.61), right heart strain (*r* = −0.03, *p* = 0.63) and total clot burden defined by the Mastora score (*r* = 0.02, *p* = 0.72).

## Discussion

The topic of body composition is an emergent field of research with extensive studies in various disorders.^8–11^ Of note, there are various possible applications and interesting prognostic implications of VAT quantification throughout many fields of clinical care.^8–10^ Importantly, VAT calculations are a by-product of cross-sectional imaging and can easily be estimated.

One great concern is a great variation of VAT calculation throughout published studies. A common approach is based on the umbilical level, which should show the highest amount of fat area.^11^ This results in higher threshold values for this level as reported by Doyle et al.: 163.8 cm² in male patients and 80.1 cm² in female patients.^21^ These values were used and tested in oncologic patients. The present study used a validated threshold of 100 cm², which was employed in patients with COVID-19.^12^

One has to consider the differences between oncologic to emergency patients in regard of constitution and co-morbidities. Clearly, larger, multicentric patient samples are needed to reliably estimate possible cut-offs to define visceral obesity for patients with PE in clinical routine.

Yet, thoracic CT for diagnosis of PE does not cover the L3 level. One reason for this study was therefore to calculate VAT on the vertebra level L1. However, VAT calculation derived from thoracic CTs did not harbour prognostic relevance in patients with PE.

The present analysis identified a high frequency of visceral obesity in patients with acute PE. A similar study on COVID-19 patients reported a frequency of 69.5% of patients with visceral obesity using the same threshold value than the present study.^12^ In another study based on a Chinese population of COVID-19 patients, the frequency was slightly lower.^22^

Due to the present COVID-19 pandemic, several authors have tried to establish thoracic CT to identify possible associations between mortality and amount of visceral fat areas in this important infectious disorder.^11–14^

The reported results showed associations of VAT with unfavourable outcomes. It was reported that an increase of visceral fat area by ten square centimetres was associated with a 1.37-fold higher likelihood of ICU treatment and a 1.32-fold higher likelihood of mechanical ventilation in patients with COVID-19.^14^ In another study based on 378 COVID-19 patients from the US, high VAT was associated with mortality in a multivariate analysis with a hazard ratio of 1.97.^12^ Importantly, the BMI was not associated with mortality and consequently, VAT provided more prognostic relevance compared to the established body parameter.^12^

Regarding body composition parameters, only one study elucidated the effect of psoas muscle areas to predict in-hospital mortality in patients with acute PE.^23^ The authors could show that psoas muscle area was significantly associated with in-hospital mortality with a reported odds ratio of 0.259, whereas established factors including heart rate and PESI score were not associated with mortality.^23^

The present results should indicate that visceral fat areas might not be a useful parameter in patients with PE and other body composition features should be further elucidated. The complex mechanisms in PE with cardiovascular insufficiency and shock are far more important in this disorder.

The present analysis has several limitations. Firstly, it is a retrospective study with possible inherent bias. Secondly, the patient sample is relatively small caused by the single centre design. There might be some selection bias resulting in the relatively high mortality rate of our patient sample compared to similar studies,^24^ which can have an influence on the presented results. Third, the VAT quantification was performed by one reader. However, the analysis was performed semi-quantitatively and should not harbour relevant reader bias. Fourth, it should be emphasised that the measurement of VAT at L1 level is influenced by the inspiration of the patients with consequently different localisation of the parenchymal organs.

## Conclusion

Visceral obesity is frequent in patients with acute PE. VAT is not associated with 30 days mortality in patients with acute PE.
